# Factors influencing day surgery patients’ quality of postoperative recovery and satisfaction with recovery: a narrative review

**DOI:** 10.1186/s13741-019-0115-1

**Published:** 2019-05-22

**Authors:** Maria Jaensson, Karuna Dahlberg, Ulrica Nilsson

**Affiliations:** 1 School of Health Sciences, Faculty of Medicine and Health, Örebro University, 70182 Örebro, Sweden; 2 Division of Nursing, Department of Neurobiology, Care Sciences, and Society, Karolinska Institute and Perioperative Medicine and Intensive Care, Karolinska University Hospital, Stockholm, Sweden

**Keywords:** Ambulatory surgical procedures, Postoperative period, Preoperative period, Patient satisfaction, Quality of healthcare

## Abstract

The aim of healthcare services is to provide a high quality of care. One way to ensure that this aim has been fulfilled is to assess patients’ satisfaction with their care. Although satisfaction is a complex concept, it is an important outcome in perioperative care. The objective of this paper is to discuss and reflect on factors that can affect patients’ quality of postoperative recovery and satisfaction with recovery after day surgery. Involving patients in shared decision-making (SDM) and providing sufficient preoperative and postoperative information can improve their satisfaction. It is important to assess whether patients experience poor recovery, which can be both distressing and dissatisfying. We suggest that patients’ age, sex, mental health status, and health literacy (HL) skills should be assessed preoperatively, since these factors seem to have a negative impact on patients’ postoperative recovery. Identifying factors that have a negative impact on patients’ quality of postoperative recovery and satisfaction with recovery after day surgery will assist healthcare professionals in supporting vulnerable patients, such as those with limited HL and poor mental health. Treating patients with respect and dignity and providing SDM can increase their quality of postoperative recovery and satisfaction with recovery.

## Background

The paradigm in patient care has shifted towards greater involvement of patients in decisions regarding planned and received care. Thus, the patient is considered to be an equal partner in terms of shared decision-making (SDM) for his or her care (Ekman et al., 2011; Kitson et al., 2013). As patients want to be involved in their own care, SDM can increase patient satisfaction (Sepucha et al., 2018; Dwamena et al., 2012; Flierler et al., 2013; Wolf et al., 2008; Suhonen et al., 2012; Suhonen et al., 2007; Hwang et al., 2014). Studies show that patients have trust in the healthcare system (Dahlberg et al., 2018a); nevertheless, it is important to meet the expectations and needs expressed by patients. In order to implement SDM and enter into a partnership with the patient, healthcare providers must be aware of factors that may be associated with poorer postoperative recovery; this knowledge will allow them to prevent such a result, if possible, and to provide support and advice to the patient and next of kin.

The objective of this narrative review is to identify factors that can affect patients’ quality of postoperative recovery and satisfaction with recovery after day surgery.

### The satisfied day surgery patient

The concept of satisfaction is complex and can be interpreted differently depending on individual beliefs (Berkowitz, 2016). Attempts have been made to define and operationalize patient satisfaction (Batbaatar et al., 2015; Singh, 1989; Wagner & Bear, 2009). In their review, Batbaatar et al. point out the following aspects of patient satisfaction: patients’ emotional evaluation, based on expectations; the association patients make between their expectations of and their experiences with healthcare; and patients’ evaluation of their care as a whole (Batbaatar et al., 2015). In a concept analysis of patient satisfaction, four attributes were identified: the provider’s attitude, the provider’s technical competence, the accessibility of healthcare, and the efficacy of healthcare. The authors also suggested that demographic characteristics such as age and sex as well as personality traits could have both positive and negative impacts on patients’ satisfaction (Ng & Luk, 2019). The concept of patient satisfaction has been suggested to be an indicator of the quality of care from the patient’s perspective (Cleary & McNeil, 1988; Farley et al., 2014; Chow et al., 2009), although some studies have failed to associate satisfaction with the quality of care (Farley et al., 2014). In other studies, patient experience has been treated as interchangeable with patient satisfaction (Berkowitz, 2016).

Several studies have explored patient satisfaction in the perioperative context (Lemos et al., 2009; Thurairatman et al., 2014; Virk et al., 2014; Lehmann et al., 2010; Heidegger et al., 2013). Different questionnaires have been used to measure patient satisfaction, which makes it difficult to interpret and compare findings (Heidegger et al., 2013; Gill & White, 2009). Even so, many clinical trials use patient satisfaction as an outcome (Heidegger et al., 2013). Despite the complexity and lack of consensus regarding how to interpret and measure patient satisfaction, we consider it to be an important factor in the provision of good quality care. We suggest that satisfaction can be seen as an indicator of quality of care and that patients’ experiences with and expectations of their care can influence their overall level of satisfaction.

Day surgery is increasing both nationally and internationally, mainly because it is a safe, efficient, and cost-effective level of care (Warner et al., 1993; Majholm et al., 2012; Mathis et al., 2013; Darwin & Chung, 2013) that is requested by patients (Mottram, 2012). Undergoing day surgery puts demands on patients to self-manage their postoperative recovery at home (Dahlberg et al., 2018a; Odom-Forren et al., 2017; Berg et al., 2013). Many patients prepare themselves preoperatively at home, with the goal of having the best possible surgical outcome (Dahlberg et al., 2018a; Berg et al., 2013). They have expectations about not living with pain and discomfort, being able to perform all household chores or certain tasks at their workplace again without feeling pain, and being able to eat all kinds of food again after surgery (Svensson et al., 2016). Patients have different strategies to prepare themselves emotionally and physically for surgery, such as exercising, preparing housework, arranging for support, and obtaining information and knowledge about the surgery and recovery (Dahlberg et al., 2018a; Berg et al., 2013). During this preoperative phase, patients have expressed the importance of being included in decision-making, in partnership with healthcare providers (Berg et al., 2013). During the postoperative phase, increased patient satisfaction is related to having pain under control (Lemos et al., 2009; Heidegger et al., 2013; Mitchell, 2015), receiving appropriate postoperative information (Lemos et al., 2009; Mitchell, 2015; Fung & Cohen, 2001), sharing in decision-making, and being treated with respect and dignity (Flierler et al., 2013; Heidegger et al., 2013; Berning et al., 2017).

Later in this paper, we will provide a more in-depth discussion of factors presented in the literature that can affect patient quality of postoperative recovery and satisfaction with recovery after day surgery.

#### Preoperative assessment and information

The purpose of a preoperative assessment (POA) is to provide safe care for the patient (Prabhakar et al., 2017) and to predict and prevent complications during surgery and early postoperative recovery (Prabhakar et al., 2017; Riggs et al., 2017). The POA involves identifying the patient’s medical problems, determining whether there is a need for additional diagnostic testing and determining the level of risk for the patient, both during and after surgery (Riggs et al., 2017; Allison & George, 2014). Healthcare providers should also respond to the patient’s expectations, requests, and needs during the POA (Prabhakar et al., 2017; Mondloch et al., 2001).

A study investigating patient satisfaction with the POA found that 80% (*n* = 221/275) of the respondents indicated that the POA prepared them for hospital admission (Fraczyk & Godfrey, 2010). Studies investigating patients’ perspectives regarding when and where a POA should be performed have yielded different results (Lemos et al., 2009; Fraczyk & Godfrey, 2010). In one study, day surgery patients seemed to be more satisfied if they visited an anaesthesia clinic prior to surgery (Lemos et al., 2009). However, another study showed that as many as 96% of patients (*n* = 269/275) were satisfied with their POA, regardless of whether it was performed through only a telephone call, a telephone call followed by an appointment, a meeting at a separate outpatient appointment after the original outpatient appointment, or a meeting at the original outpatient appointment at the hospital (Fraczyk & Godfrey, 2010).

The provision of sufficient preoperative information can preoperatively increase patient satisfaction (Mitchell, 2015; Fung & Cohen, 2001). The mode and timing of the provision of preoperative information require careful consideration of patient participation, and the information strategies used should be optimal. Some patients report experiencing heightened stress and anxiety while visiting the hospital for preoperative information and assessment (Gilmartin, 2004; Kindler et al., 2005).

The results from a randomized controlled trial revealed that providing personalized information—that is, an empathic response to emotions, instead of routine, standardized information about hospital procedures and surgical preparation—could reduce patient anxiety. The intervention group reported significantly reduced anxiety levels and lower levels of postoperative pain and were more satisfied with the care received (Pereira et al., 2016). Preoperative anxiety can affect as many as 60–80% of surgical patients (Pokharel et al., 2011) and is associated with the patient’s sex, fear of complications, previous surgical experience, and access to preoperative information (Pokharel et al., 2011; Mulugeta et al., 2018). Preoperative information is therefore an important factor in the POA process (Lemos et al., 2009; Mitchell, 2015).

One barrier to information transfer occurs when the process does not meet the patients’ individual needs (Gilmartin, 2004). Due to a lack of time, the conversation during a preoperative meeting focuses mainly on biomedical issues, with little focus on psychosocial aspects (Gilmartin, 2004; Kindler et al., 2005). A lack of sufficient information can cause uncertainty and anxiety for the patient (Dahlberg et al., 2018a; Berg et al., 2013), and some patients may also feel unsure regarding whether they have understood the information (Svensson et al., 2016). Conflict exists between what and how much preoperative information should be included during the meeting, and what a patient may be able to comprehend before a day surgery. Providing patients with written preoperative information during the POA visit can enable them to read and absorb facts about the procedure prior to hospitalization. Patients can also discuss written information with their family or friends, in order to elicit support and reduce their own anxiety (Gilmartin, 2004).

It is challenging for a healthcare provider to meet patients’ expectations regarding the amount and type of information needed (Sibbern et al., 2017), since patients have different coping styles (Mitchell, 2000). Some patients want detailed information, while others prefer concise information (Wongkietkachorn et al., 2018). A recent randomized controlled trial attempted to address these differences by providing the intervention group with needs-based education, with different levels of information being assessed prior to the preoperative meeting with the physician. Patients received both verbal and written information; the difference between the patients in the intervention group and those in the control group lay in the amount of verbal information provided. Prior to the preoperative meeting, the patients filled out a questionnaire on how much information (i.e. none, concise, or detailed) was desired on a number of topics; the topics included were disease information, procedural detail, possible complications (this verbal information was mandatory), and patient behaviour (i.e. “What should I do?”) (Wongkietkachorn et al., 2018). The results showed that needs-based education significantly reduced patient anxiety and increased patient satisfaction both directly after the education and postoperatively. Needs-based information also reduced the time spent by the physician in educating the patients (Wongkietkachorn et al., 2018). This novel study shows that SDM can have an impact on patient satisfaction; however, this study was limited in that the sample only included patients undergoing day surgery under local anaesthesia.

#### Age

Age is often analysed as a predictor for satisfaction; in fact, it appears that a patient’s age is associated with both satisfaction and quality of recovery (Lemos et al., 2009; Myles et al., 2000; Capuzzo et al., 2007). Investigations on the overall level of satisfaction with care have noted that older patients tend to be more satisfied; however, the cut-off age varies with studies defining “older” patients as being over 44 years (Lemos et al., 2009), over 65 years (Myles et al., 2000), and over 70 years of age (Capuzzo et al., 2007). Overall, older patients are more likely to be satisfied with perceived SDM during their preoperative visit (Flierler et al., 2013). Another association depending on age has also been noted: older patients report better quality of postoperative recovery compared with younger patients (Jaensson et al., 2018; Stessel et al., 2015). The aetiology for the difference in satisfaction between younger and older patients is unclear, however, which makes the interpretation and generalization of these results difficult. Further investigation is needed on the association of age with satisfaction, preferably with the primary aim of investigating whether patient satisfaction differs between different age groups.

#### Health literacy

Patients’ inability to recall the postoperative information that has been provided presents another challenge for healthcare providers (Berg et al., 2013; Greenslade et al., 2010). This inability may be a result of anaesthesia and the surgery itself; however, it may also be due to a lack in patients’ health literacy (HL) skills.

The concept of HL relates to one’s ability to assess, understand and use information in order to maintain or improve one’s health (Rudd, 2015). Limited HL skills are associated with problems taking medication according to a prescription (Berkman et al., 2011), difficulty understanding health information (Berkman et al., 2011), overall poorer health status (Berkman et al., 2011; Halleberg Nyman et al., 2018), poorer postoperative recovery (Halleberg Nyman et al., 2018), and a longer stay for patients undergoing major surgery (Wright et al., 2018). Subgroups with increased risk of limited HL include older persons (Berkman et al., 2011) and people with low income (Jessup et al., 2017) and a low educational level (Protheroe et al., 2017). The results from a meta-analysis on the prevalence of HL among surgical patients (*n* = 18,895) showed that 31.7% had limited HL (Roy et al., 2019). Similarly, a study reported that 39.4% of day surgery patients had limited HL skills, which were found to be associated with poorer quality of postoperative recovery (Halleberg Nyman et al., 2018). Another study showed that patients with adequate HL reported higher satisfaction scores than those with inadequate HL (Yim et al., 2018). However, it has also been reported that day surgery patients who are satisfied with their entire surgical healthcare experience tend to have a lower level of academic education than those who are not totally satisfied (Lemos et al., 2009). The reason for this contradiction remains unclear. There is also a lack of evidence on the impact of HL on patient outcomes in the perioperative context (De Oliveira Jr. et al., 2015).

In addition, no evidence exists on whether or not current healthcare services meet the needs of those with limited HL skills. Patient information and hospital websites require further development to meet different HL needs (Keinki et al., 2018; Kim & Xie, 2017). Furthermore, it is extremely important that healthcare providers be aware of the problem when informing the patient, as this will allow them to take appropriate steps such as using plain language when providing preoperative information (Elgin, 2018; Ross, 2013). It is also notable that people with limited HL skills may ask fewer questions about medical issues than those with adequate skills (Roh et al., 2018), possibly due to poor comprehension of medical information (De Oliveira Jr. et al., 2015). As a result, patients with limited HL skills may take on a more passive role in SDM (Roh et al., 2018).

#### Mental health

Preoperative psychological status has been recognized as an influencer of postoperative recovery relatively recently, although it is now a well-discussed topic on public forums. Preoperative anxiety and/or depression is an important predictor of dissatisfaction with surgical outcome (Ali et al., 2017) and can affect patient dissatisfaction for up to 2 years after surgery (Adogwa et al., 2014). Aspari and Lakshman (2018) have stated that mental health is one of the most overlooked factors in daily clinical practice and have reported that poor mental health has a negative effect on postoperative recovery in terms of pain perception, return to work, and quality of life. It has also been reported that low preoperative mental health is associated with poorer postoperative recovery, which may include poorer postoperative sleep quality, lacking a general feeling of well-being, greater suffering from postoperative pain, difficulty concentrating, and difficulty returning to work or to usual home activities (Nilsson et al., 2019). Furthermore, there appears to be a gender difference in terms of psychological status, as women are reported to suffer from higher rates of poor mental health than men preoperatively (Nilsson et al., 2019). Yet gender similarities have also been reported regarding the preoperative emotional state of patients undergoing day surgery (Nilsson et al., 2009). Assessment and documentation of the patient’s preoperative mental health should be included in the standard POA. If the patient’s mental health status requires it, formal counselling must then be instituted in order to psychologically prepare and support the patient to face surgery. Such mental preparation is as important as a patient’s physical preparation for surgery (Aspari & Lakshman, 2018). Using personalized information and providing an empathic response to the patient’s emotions can be helpful in supporting vulnerable patients (Kindler et al., 2005; Pereira et al., 2016).

#### Gender/sex

In this paper, the term *sex* is used to refer to the biology of human and animal subjects, and the term *gender* is used to refer to an individual’s self-identity and/or social representation (Torgrimson & Minson, 2005).

Earlier studies have shown that sex affects postoperative recovery, with women being prone to poorer recovery (Myles et al., 2001; Myles et al., 1997; Buchanan et al., 2011; Buchanan et al., 2009). It is likely, therefore, that women will be more dissatisfied than men with the quality of care after day surgery (Myles et al., 2000; Teunkens et al., 2017). Genetics and biology differ between the sexes; however, personalized medicine takes sex into account. For example, women are more prone to post-discharge nausea and vomiting and therefore receive appropriately prophylactic medication (Apfel et al., 2012). Gender role expectations and gender stereotypes—that is, beliefs regarding how men and women should act or behave (Koenig, 2018)—can also play a part in postoperative recovery. Koenig (2018) has reported that people believe men tend to show less emotion than women and that gender role stereotypes may be less relevant in older age groups (Koenig, 2018). This finding may help to explain why women report more dissatisfaction and older patients report more satisfaction with their care. Few studies have been published on gender similarities in postoperative recovery. However, one study measuring recovery over a 14-day period after day surgery found no difference in the postoperative recovery of men and women (Jaensson et al., 2018).

#### Follow-up and support after discharge

There is no consensus regarding when and how follow-up after day surgery should be performed (Discharge process and criteria, 2016). In general, a telephone call is made 1 day after the surgery (Stomberg et al., 2013; Segerdahl et al., 2008). There is a growing body of research on the use of digital follow-up tools (Semple et al., 2015; Armstrong et al., 2017; Warren-Stomberg et al., 2016; Nilsson et al., 2016; Williams et al., 2018), including digital tools that patients find easy to use (Semple et al., 2015; Dahlberg et al., 2016; Jaensson et al., 2015; Debono et al., 2016) and that have a positive effect on postoperative recovery (Jaensson et al., 2017). Digital follow-up has been described by patients as increasing their feeling of safety and helping them to not feel alone after day surgery (Dahlberg et al., 2018a). Providing patients with digital follow-up that includes some sort of measurement of the postoperative recovery process (Jaensson et al., 2015) may also address patients’ need for postoperative information by implicitly informing them of issues, discomforts, and symptoms that can happen during postoperative recovery (Jaensson et al., 2017).

To transition postoperative care towards a more person-centred approach, we recommend involving patients in decisions regarding when they have contact with healthcare services instead of using standardized timing, as the latter can result in contact that is either too often or not often enough (Dahlberg et al., 2018b). Allowing patients to decide whether and when to be contacted after discharge seems to increase the quality of their postoperative recovery. This positive effect on recovery may be due to an increase in patients’ self-efficacy that results from self-management in deciding when contact with the healthcare service is needed (Dahlberg et al., 2018a; Barnason et al., 2003). It is also known that patients’ satisfaction after anaesthesia and surgery can be enhanced by having nurses be easily accessible (Berning et al., 2017). Furthermore, having patients undergo a personalized e-health programme to manage their recovery expectations and to obtain postoperative guidance tailored to their needs can enhance postoperative recovery (van der Meij et al., 2018).

## Conclusion

Postoperative recovery after day surgery can be improved not only by addressing comorbidities, health status, and type of surgery but also by taking into account the patient’s HL, age, and mental health status; providing relevant preoperative and postoperative information; and establishing an accessible support system. Valid and reliable instruments should be used to preoperatively screen for HL and mental health status. Although these issues can be delicate, it is important for surgeons to be aware of contributory factors to an optimal recovery process, as surgeons are the first to meet the patient in the surgical process. Anaesthetists and nurses at the day surgery unit also play an important role in detecting factors that can negatively influence patients’ postoperative recovery. Therefore, perioperative healthcare professionals should be formally trained to identify and address these factors. This knowledge will allow healthcare professionals to support vulnerable patients such as those with limited HL and poor mental health. Treating patients with respect and dignity as individuals and engaging patients in SDM can increase both quality of postoperative recovery and satisfaction with recovery. Further research is called for to measure the effect of this suggested preoperative screening and to identify other factors that influence the quality of recovery and patient satisfaction with recovery.
